# Comparison of health-related quality of life among patients using atypical antipsychotics for treatment of depression: results from the National Health and Wellness Survey

**DOI:** 10.1186/1477-7525-10-81

**Published:** 2012-07-17

**Authors:** Iftekhar Kalsekar, Jan-Samuel Wagner, Marco DiBonaventura, Jay Bates, Robert Forbes, Tony Hebden

**Affiliations:** 1 Bristol-Myers Squibb, 777 Scudders Mill Road, Plainsboro, NJ, 08536, USA; 2 Health Sciences Practice, Kantar Health, 11 Madison Avenue, 12th Floor, New York, NY, 10010, USA; 3 Otsuka Pharmaceutical Development & Commercialization, Inc., Princeton, NJ, USA

## Abstract

**Background:**

Use of atypical antipsychotics (AA) in combination with an antidepressant is recommended as an augmentation strategy for patients with depression. However, there is a paucity of data comparing aripiprazole and other AAs in terms of patient reported outcomes. Therefore, the objective of this study was to examine the levels of HRQoL and health utility scores in patients with depression using aripiprazole compared with patients using olanzapine, quetiapine, risperidone and ziprasidone.

**Methods:**

Data were obtained from the 2009, 2010, and 2011 National Health and Wellness Survey (NHWS), a cross-sectional, internet-based survey that is representative of the adult US population. Only those patients who reported being diagnosed with depression and taking an antidepressant and an atypical antipsychotic for depression were included. Patients taking an atypical antipsychotic for less than 2 months or who reported being diagnosed with bipolar disorder or schizophrenia were excluded. Patients taking aripiprazole were compared with patients taking other atypical antipsychotics. Health-related quality of life (HRQoL) and health utilities were assessed using the Short Form 12-item (SF-12) health survey. Differences between groups were analyzed using General Linear Models (GLM) controlling for demographic and health characteristics.

**Results:**

Overall sample size was 426 with 59.9% taking aripiprazole (n = 255) and 40.1% (n = 171) taking another atypical antipsychotic (olanzapine (n = 19), quetiapine (n = 127), risperidone (n = 14) or ziprasidone (n = 11)). Of the SF-12 domains, mean mental component summary (MCS) score (p = .018), bodily pain (p = .047), general health (p = .009) and emotional role limitations (p = .009) were found to be significantly higher in aripiprazole users indicating better HRQoL compared to other atypical antipsychotics. After controlling for demographic and health characteristics, patients taking aripiprazole reported significantly higher mean mental SF-12 component summary (34.10 vs. 31.43, p = .018), bodily pain (55.19 vs. 49.05, p = .047), general health (50.05 vs. 43.07, p = .009), emotional role limitations (49.44 vs. 41.83, p = .009), and SF-6D utility scores (0.59 vs. 0.56, p = .042).

**Conclusions:**

Comparison of patients taking aripiprazole with a cohort of patients using another AA for depression demonstrated that aripiprazole was independently associated with better (both statistically and clinically) HRQoL and health utilities.

## Background

Major depressive disorder (MDD) is a recurrent mental illness with a lifetime prevalence of approximately 16% in the United States (US) [1]. Marked by depressed mood and the inability to experience pleasure from activities, MDD is associated with premature mortality due to suicide and comorbidities such as cardiovascular and respiratory disease [2]. Donohue & Pincus (2007) estimated the healthcare costs of depression in the US were $26 billion per year and indirect costs (due to lost productivity in the workplace) were $52 billion per year [3]. Kessler et al. (2003) found the majority of patients who reported experiencing MDD in the past 12 months also experienced moderate to severe role limitations at home or work, or in their relationships (as measured by the Sheehan Disability scale) [1]. By 2020, it is estimated depressive disorders will rank as the second most costly group of diseases globally, in terms of disability adjusted years lost [3].

Patients with depression also report significant impairments in health-related quality of life (HRQoL). Results from the Medical Outcomes Study showed patients with depression had mental component summary (MCS) scores approximately 1 standard deviation below the general US population [4]. Remission of symptoms has been found to lead to improved quality of life [4].

Although it is important to note that treating depression is generally cost-effective, less than 1 out of 3 patients with clinical depression achieve remission after completing a course of first-line therapy [5]. In fact, as widely cited from the STAR*D study, 2 out of 5 patients with clinical depression will not respond to depression treatment, even after they have completed a course of fourth-line therapy [5].

Because of inadequate response to treatment, newer molecules such as atypical antipsychotics, which were not included in the STAR*D study, have been increasingly used in clinical practice. A number of randomized clinical trials have demonstrated the beneficial effects of olanzapine, quetiapine, risperidone and aripiprazole [6-8] as augmentation to current antidepressants. Indeed, a recent article by the World Psychiatric Association suggests that the best evidence for antidepressant augmentation resides with atypical antipsychotics [9]. However, literature reviews by Chen et al. (2011) and Pae et al. (2011) produced no direct head-to-head comparisons of atypical antipsychotics as augmentation treatments for MDD and noted the need for pharmacoeconomic comparisons of atypical antipsychotics [7,10]. Therefore, the objective of this study is, in a real world setting, to examine the levels of HRQoL and health utility scores in patients with depression using aripiprazole compared with patients using olanzapine, quetiapine, risperidone and ziprasidone.

## Methods

### Data source

This study used data from the 2009, 2010, and 2011 US National Health and Wellness Survey (NHWS) databases (Kantar Health, New York, NY). Data from these surveys were collected between January 1, 2009 and March 1, 2011. The NHWS is a cross-sectional, internet-based survey of adults (aged 18+). Using a stratified random sampling framework, potential respondents are recruited through internet panels such that the demographic composition of the NHWS is identical to that of the general population, as measured by the US Census. The reliability and validity of the NHWS, particularly as it relates to disease prevalence, has been assessed in prior research [11-13]. These studies have compared data collected from the NHWS and various sources including the US Census Bureau’s Current Population Survey, National Health Interview Survey, National Health and Nutrition Examination Survey, and the Medical Expenditure Panel Survey [11-13].

All participants of the NHWS agreed to participate in the survey explicitly and were awarded nominal incentives for their participation. The NHWS is approved each year by Essex Institutional Review Board (Lebanon, NJ). Of 1,157,205 who were invited to participate (from January 1, 2009 to March 1, 2011), 250,849 responded (21.68% response rate). Of those who responded, 175,000 (69.76%) met the study inclusion criteria (gave informed consent and were aged 18 or over) and completed the survey.

### Sample and procedure

Because the NHWS is fielded separately each year, it is possible for a respondent to complete the survey in multiple years (e.g., the same respondent could have completed the NHWS in 2009 and 2010). In such cases, only data from the most recent time point was kept and so a total of 156,582 unique respondents were available. Respondents were included in the study if they self-reported a diagnosis of depression, were taking both an antidepressant and an atypical antipsychotic medication for their depression, were taking all their depression medications for at least two months, and had complete data on all study covariates. Respondents were excluded if they self-reported a diagnosis of schizophrenia or bipolar disorder.

### Measures

#### Main independent variable

##### Atypical antipsychotic use

The primary independent variable was a dichotomous variable comparing patients taking aripiprazole and an antidepressant versus patients taking another atypical antipsychotic (olanzapine, quetiapine, risperidone, or ziprasidone) and an antidepressant. For a complete list of antidepressants assessed in the NHWS see Additional file 1.

#### Outcomes

##### Health-related quality of life (HRQoL)

To measure HRQoL, every respondent was asked to complete version 2 of the Medical Outcomes Study Short Form 12 (SF-12) questionnaire [14]. The SF-12 is a generic measure of HRQoL, which assesses 8 health domains: physical functioning, physical role limitations, bodily pain, general health perceptions, vitality, social functioning, emotional role limitations, and mental health [14]. The health domains are summarized by two summary scores: the mental component summary (MCS) and physical component summary (PCS), which are both standardized to the US general population (Mean = 50, Standard Deviation = 10). Both domain scores and summary scores vary between 0 and 100; where higher scores indicate greater health. Previous findings suggest the SF-12 instrument is sensitive to remission of depressive symptoms [15] and response to treatment among depression patients [16]. Prior research has suggested that differences of 3 points in MCS and PCS are considered clinically meaningful [17].

##### Health utility

The SF-6D, which is a preference-based health utility index calculated from the SF-12 items, was also reported [18]. Scores vary from 0.29-1.00, where 1.00 indicates perfect health. Previous findings indicate the SF-6D is sensitive to remission of depressive symptoms, with 0.11 quality-adjusted life years gained over a two year period with remission of symptoms [4]. Differences of 0.03 points in the SF-6D health utility are considered clinically meaningful [19].

#### Covariates

##### Demographic and health characteristics

The following information was assessed for all respondents: age, gender, ethnicity/race, marital status, educational attainment, household income, employment status, insurance coverage, body mass index (BMI; measured as kg/m^2^), exercise behavior, alcohol consumption, tobacco smoking behavior, antidepressant use, type of prescriber, year surveyed, time since diagnosis, and comorbidity burden (using the Charlson Comorbidity Index) [20].

### Statistical analyses

Bivariate analyses were conducted to describe and compare the demographic and health characteristics of patients being treated with aripiprazole versus other atypical antipsychotics. For categorical variables, chi-square tests were used to determine significant differences, while t-tests were used for continuous variables.

Although our original intent was to compare aripiprazole with each individual atypical antipsychotic, this was not possible due to small sample sizes of some of the comparators. As a result, we focused primarily on the comparison between aripiprazole and all other atypical antipsychotics pooled together. These main analyses compared those using aripiprazole with those using all other atypical antipsychotics on all HRQoL and health utility scores using t-tests. These differences were also examined using General Linear Models (GLM), controlling for the covariates described above in order to mitigate the effect of selection bias (i.e., the systematic reasons why a patient may be prescribed one atypical medication over another which also may influence HRQoL). Sub-analyses were also conducted to compare users of aripiprazole with users of each individual atypical antipsychotic, though only the comparison with quetiapine had sufficient sample size for any meaningful interpretation. Differences between the groups were first made on an unadjusted level using t-tests. Subsequent GLMs were then conducted to compare for group differences while controlling for the covariates mentioned above.

Adjusted means, which provide the mean HRQoL and utility scores when all covariates are set at the sample mean, were obtained through a least-squares algorithm for all GLMs (both in the main analysis and the sub-analysis). All models controlled for the following variables (reference variables are marked by an *): age (in years), gender (female*, male), ethnicity/race (non-Hispanic white*, non-Hispanic black, Hispanic, other), marital status (married or living with a partner, all else*), region (Northeast*, South, West, Midwest), household income (less than $25,000*, $25,000 to ≤ $50,000, $50,000 to ≤ $75,000, $75,000 or more), insurance coverage (yes, no*), year surveyed (2009, 2010*, 2011), exercise behavior (none in the past month*, 1 to 9 times in the past month, 10 or more times in the past month), alcohol consumption (none in the past month*, once or more in the past month), BMI (underweight or normal weight*, overweight, obese), comorbidity burden (Charlson Comorbidity Index as a continuous variable), prescriber type (psychiatrist, all else*), time since diagnosis (less than 4 years*, 5 to 10 years, 11 or more years), selective serotonin reuptake inhibitor use (yes, no*), selective norepinephrine reuptake inhibitor (yes, no*), tricyclic antidepressant (yes, no*), other antidepressant use (yes, no*).

Sensitivity analyses were also conducted to assess the robustness of the findings. Rather than entering all covariates simultaneously in the model, a series of hierarchical multiple regressions were conducted to predict all summary and domain HRQoL scores. For each outcome, the following process was initiated separately. In the first step, all demographic variables were entered (age, gender, ethnicity, region, marital status, household income, health insurance) into a multiple regression model. For the second step, only the significant variables from the prior step as well as all health characteristic information (smoking behavior, exercise behavior, alcohol consumption, BMI, Charlson Comorbidity Index, prescriber type, time since diagnosis, and concomitant treatments) were entered into a multiple regression model. For the third step, all significant variables from the prior step, as well as atypical antipsychotic use (aripiprazole versus other atypical antipsychotics) were entered into a general linear model. Only the adjusted means from the third, and final model, are reported. All analyses were conducted in SAS v9.1 (Cary, NC) and the a priori cutoff for statistical significance was p<.05.

## Results

### Sample characteristics

**Figure 1:**
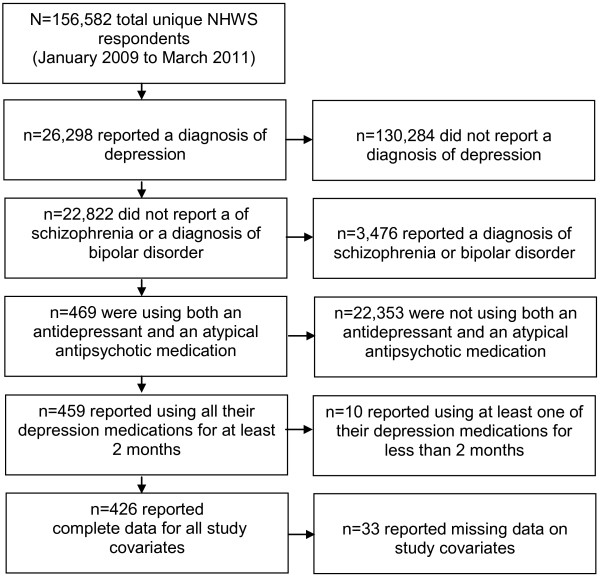
Study flowchart.

The study flowchart is shown in Figure 1. A total of 426 respondents met all study inclusion and exclusion criteria. Of this group, 255 respondents reported taking aripiprazole (59.86%) and 171 respondents reported taking another atypical antipsychotic (40.14%). Of those taking another atypical antipsychotic, 11.11% were taking olanzapine (n = 19), 74.27% were taking quetiapine (n = 127), 8.19% were taking risperidone (n = 14), and 6.43% were taking ziprasidone (n = 11). Compared to other atypical antipsychotic users, respondents on aripiprazole were generally similar demographically (see Table 1). However, respondents taking aripiprazole were significantly more likely to be from the South, retired, on disability, and more likely to currently possess health insurance. These respondents were also less likely to currently smoke (*p*<.05). Respondents on aripiprazole were similar to other atypical antipsychotic users with respect to their prescribing physician and the years they had been diagnosed with depression (see Table 2). However, aripiprazole users were significantly more likely to be concomitantly treated with a selective norepinephrine reuptake inhibitor (*p*<.05).

**Table 1:** Demographic and health characteristic differences between patients taking aripiprazole or another atypical antipsychotic to treat their depression

**Variable**		**Aripiprazole (n = 255)**		**Other atypical (n = 171)**		**χ**^**2**^	**p**
		**n**	**%**	**n**	**%**		
Male		110	**43.14%**	81	**47.37%**	0.74	0.391
Ethnicity							
	White	194	**76.08%**	129	**75.44%**	0.02	0.880
	Black	31	**12.16%**	19	**11.11%**	0.11	0.741
	Hispanic	14	**5.49%**	14	**8.19%**	1.13	0.289
	Other	16	**6.27%**	9	**5.26%**	0.20	0.659
Married/living with partner		112	**43.92%**	68	**39.77%**	0.73	0.394
Region							
	Northeast	48	**18.82%**	36	**21.05%**	0.32	0.575
	Midwest	57	**22.35%**	50	**29.24%**	2.50	0.115
	South	98	**38.43%**	49	**28.66%**	4.48	0.035
	West	52	**20.39%**	36	**21.05%**	0.03	0.869
Some college education (or higher)		194	**76.08%**	123	**71.93%**	0.91	0.342
Annual household income							
	<$25 K	98	**38.43%**	69	**40.35%**	0.16	0.692
	$25 K to < $50 K	75	**29.41%**	53	**30.99%**	0.12	0.728
	$50 K to < $75 K	45	**17.65%**	24	**14.04%**	1.02	0.313
	$75 K or more	37	**14.51%**	25	**14.62%**	0.00	0.975
Employment status							
	Currently employed	95	**37.25%**	64	**37.43%**	0.00	0.971
	Unemployed	55	**21.57%**	54	**31.58%**	5.18	0.023
	On disability	20	**7.84%**	7	**4.09%**	2.73	0.099
	Retired	41	**16.08%**	11	**6.43%**	10.49	0.001
On health insurance		230	**90.20%**	138	**80.70%**	7.13	0.008
Currently smoke		83	**32.55%**	78	**45.61%**	7.35	0.007
Exercise behavior							
	None in past month	128	**50.20%**	85	**49.71%**	0.01	0.921
	One to nine times a month	66	**25.88%**	38	**22.22%**	0.76	0.385
	Ten or more times a month	61	**23.92%**	48	**28.07%**	0.91	0.342
Currently drink alcohol		135	**52.94%**	106	**61.99%**	3.46	0.063
Body mass index							
	Normal	40	**15.69%**	41	**23.98%**	4.32	0.038
	Overweight	65	**25.49%**	46	**26.90%**	0.10	0.746
	Obese	147	**57.65%**	84	**49.12%**	2.99	0.084
		**Mean**	**SD**	**Mean**	**SD**	**t**	**p**
Age		**47.87**	12.41	**46.22**	11.1	1.40	0.162
Charlson comorbidity index		**0.94**	1.78	**1.16**	1.78	−1.47	0.142

**Table 2:** Depression treatment history differences between patients taking aripiprazole or another atypical antipsychotic to treat their depression

**Variable**		**Aripiprazole (n = 255)**		**Other atypical (n = 171)**		**χ**^**2**^	**p**
		**n**	**%**	**n**	**%**		
Psychiatrist as prescribing physician		177	**69.41%**	124	**72.51%**	0.48	0.489
Years diagnosed with depression							
	1 - 5 years	68	**26.67%**	42	**24.56%**	0.24	0.625
	6 - 10 years	55	**21.57%**	43	**25.15%**	0.72	0.395
	11 years or more	129	**50.59%**	84	**49.12%**	0.09	0.767
Concomitant treatments							
	SSRI	130	**50.98%**	103	**60.23%**	3.58	0.059
	SNRI	111	**43.53%**	43	**25.15%**	16.23	<.0001
	TCA	28	**10.98%**	21	**12.28%**	0.17	0.684
	Other	85	**33.33%**	64	**37.43%**	0.75	0.388

### Main analysis: unadjusted differences between respondents using aripiprazole and respondents using other atypical antipsychotics

In bivariate (unadjusted) comparisons, respondents using aripiprazole reported significantly higher mean levels of mental component summary (MCS) scores (34.26 vs. 32.09, p = .047, Cohen’s d = 0.20) when compared with respondents using other atypical antipsychotics, though no differences were observed on physical component summary (PCS) scores (42.05 vs. 40.14, p = .11). HRQoL was also significantly higher for patients using aripiprazole in the domains of bodily pain scores (56.18 vs. 48.83; p = .018, Cohen’s d = 0.24), general health (49.92 vs. 42.95; p = .013, Cohen’s d = 0.25), and emotional role limitations (50.05 vs. 42.98, p = .012, Cohen’s d = 0.25). Differences in mean health utility scores were marginally significant (0.57 vs. 0.54, p = .055), though they were above clinically-relevant levels (i.e., greater than or equal to 0.03).

### Main analysis: adjusted differences between respondents using aripiprazole and respondents using other atypical antipsychotics

**Figure 2:**
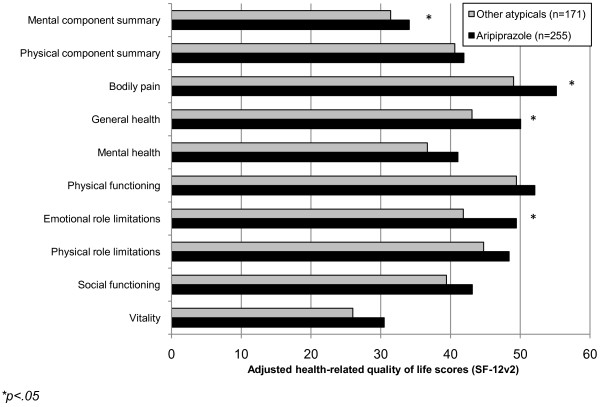
Adjusted HRQoL scores of patients using aripiprazole versus other atypical antipsychotics for depression. *p<.05.

**Figure 3:**
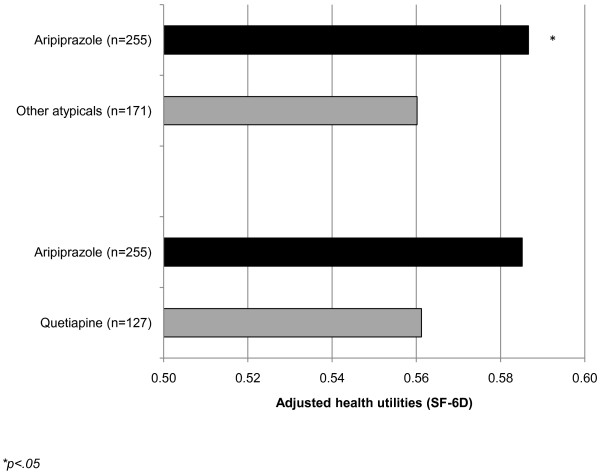
Adjusted health utility scores between patients using aripiprazole and other atypical antipsychotics. *p<.05.

After controlling for demographics and health characteristic variables, the directionality of the differences were similar to the unadjusted analysis, however the size of the differences increased (see Figure 2). Respondents using aripiprazole reported significantly higher adjusted mean levels of mental component summary (MCS) scores (34.10 vs. 31.43, p = .018). These respondents also reported significantly greater HRQoL as measured by the bodily pain (55.19 vs. 49.05, p = .047), general health (50.05 vs. 43.07, p = .009), and emotional role limitation domain scores (49.44 vs. 41.83, p = .009). Differences in adjusted mean health utility scores were also significant (0.59 vs. 0.56, p = .042) (see Figure 3).

Although the intent of these multivariable models was to mitigate the effect of selection biases when examining the differences between respondents using aripiprazole and respondents using other atypical antipsychotics, the regression output can also provide meaningful context for these differences (see Table 3). For brevity, only the details of the models predicting MCS, PCS, and health utility scores are provided.

**Table 3:** Regression estimates of all sociodemographic and depression history variables

	**b**	**MCS**		**PCS**			**Health utilities**		
		**95% LCL**	**95% UCL**	**b**	**95% LCL**	**95% UCL**	**b**	**95% LCL**	**95% UCL**
Intercept	31.754	24.302	39.205	52.049	44.581	59.517	0.653	0.570	0.735
Aripiprazole	2.673	0.460	4.886	1.321	−0.898	3.539	0.026	0.001	0.052
Age	0.150	0.056	0.245	−0.245	−0.339	−0.150	0.000	−0.001	0.001
Male	−0.569	−2.741	1.603	0.275	−1.902	2.452	−0.003	−0.028	0.023
Black	−0.506	−3.859	2.847	1.756	−1.604	5.117	0.013	−0.025	0.052
Hispanic	−3.834	−8.186	0.518	0.021	−4.341	4.383	−0.061	−0.111	−0.011
Other race/ethnicity	1.188	−3.335	5.712	−0.643	−5.177	3.891	0.014	−0.036	0.064
Married/living with partner	−4.480	−6.856	−2.104	−0.457	−2.839	1.924	−0.023	−0.050	0.003
Midwest region	−3.498	−6.645	−0.351	0.791	−2.363	3.945	−0.016	−0.051	0.018
South region	−1.439	−4.409	1.532	1.666	−1.311	4.643	0.000	−0.033	0.033
West region	0.386	−2.965	3.738	1.371	−1.988	4.730	0.011	−0.027	0.049
Some college education (or higher)	−0.193	−2.725	2.340	1.389	−1.150	3.927	−0.009	−0.037	0.019
Income: $25 K to < $50 K	2.076	−0.571	4.723	0.881	−1.772	3.534	0.014	−0.016	0.044
Income: $50 K to < $75 K	5.593	2.343	8.843	1.175	−2.082	4.432	0.055	0.018	0.092
Income: $75 K or more	3.687	0.110	7.265	3.558	−0.027	7.144	0.039	−0.001	0.078
Health insurance	−1.798	−4.891	1.296	0.644	−2.456	3.744	−0.012	−0.047	0.023
2009 survey	0.057	−2.892	3.005	−0.302	−3.257	2.653	−0.015	−0.041	0.010
2010 survey	2.393	−0.132	4.919	−0.909	−3.441	1.622	--	--	--
2011 survey	--	--	--	--	--	--	*	*	*
Currently smoke	0.526	−1.749	2.800	−3.891	−6.171	−1.612	−0.005	−0.031	0.020
Exercise 1–9 times a month	1.238	−1.436	3.911	0.599	−2.080	3.278	0.001	−0.028	0.031
Exercise 10+ times a month	1.811	−0.773	4.394	1.047	−1.542	3.636	−0.004	−0.035	0.026
Currently drink alcohol	0.329	−1.880	2.539	1.326	−0.889	3.541	0.021	−0.004	0.047
Overweight	−0.618	−3.769	2.532	−2.452	−5.610	0.706	−0.034	−0.070	0.003
Obese	−0.714	−3.565	2.138	−5.169	−8.027	−2.311	−0.055	−0.088	−0.021
Charlson comorbidity index	0.157	−0.551	0.865	−2.056	−2.766	−1.347	−0.009	−0.017	0.000
Psychiatrist as prescribing physician	−2.574	−4.936	−0.212	2.735	0.368	5.101	−0.012	−0.039	0.015
Diagnosed for 6–10 years	−0.006	−2.963	2.951	−0.450	−3.413	2.514	0.005	−0.028	0.039
Diagnosed 11 years or more	0.138	−2.542	2.817	−0.922	−3.607	1.763	0.001	−0.030	0.033
SSRI	−2.511	−5.373	0.352	1.090	−1.778	3.959	−0.009	−0.041	0.023
SNRI	−3.959	−6.979	−0.939	2.353	−0.674	5.379	−0.022	−0.056	0.012
TCA	−3.710	−7.054	−0.366	−2.941	−6.292	0.410	−0.063	−0.100	−0.025
Other treatment	−2.044	−4.358	0.270	0.493	−1.826	2.812	−0.027	−0.053	−0.001

Reference categories included: non-Hispanic white, Northeast region, income < $25 K, not exercising, normal BMI level, being diagnosed for less than 6 years.

*Health utilities were not available from respondents of the 2011 survey; as a result, the survey year 2010 served as the reference group in the health utilities model.

A sensitivity analysis was conducted using a series of hierarchical multiple regressions to determine the extent to which the exclusion of non-significant covariates influenced the observed effects. The findings were generally consistent with the main analysis. Respondents using aripiprazole reported significantly higher levels of MCS (34.28 vs. 32.06, p = .040), bodily pain (55.86 vs. 49.31, p = .025), general health (48.84 vs. 43.07, p = .007), and emotional role limitations (49.74 vs. 43.45, p = .007). However, differences in health utilities (0.59 vs. 0.57, p = .15) were not significant.

### Sub-analysis: comparing respondents using aripiprazole with respondents using individual atypical antipsychotics

**Figure 4:**
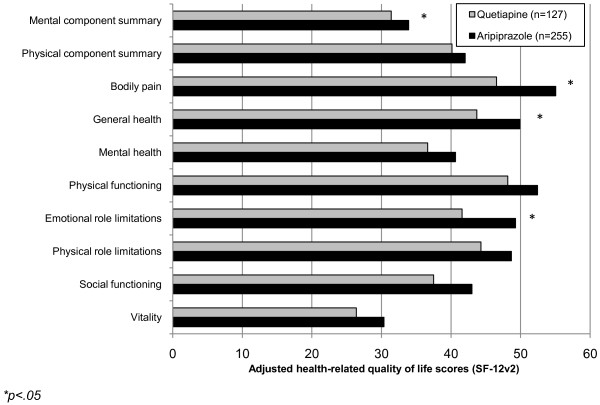
Adjusted HRQoL scores of patients using aripiprazole versus quetiapine.*p<.05.

In a sub-analysis, users of individual atypical antipsychotics were compared with users of aripiprazole. The first comparison was made with respondents using quetiapine. Similar to the main analysis, aripiprazole users were significantly more likely to be disabled (7.84% vs. 3.15%, p = .042), retired (16.08% vs. 6.30%, p = .002), insured (90.20% vs. 78.74%, p = .006), and using an SNRI (43.53% vs. 24.41%, p<.001) and significantly less likely to currently smoke (32.54% vs. 47.24%, p = .006), drink alcohol (52.94% vs. 65.35%, p = .019), and be using an SSRI (50.98% vs. 62.20%, p = .036) than users of quetiapine. In bivariate comparisons of HRQoL, aripiprazole users reported significantly higher levels of MCS (34.26 vs. 31.91, p = .041), bodily pain (56.18 vs. 46.26, p = .003), general health (49.92 vs. 43.78, p = .045), mental health (41.57 vs. 36.81, p = .048), and emotional role limitations (50.05 vs. 42.62, p = .018) than patients using quetiapine. Although all other summary and domain scores were greater among patients using aripiprazole, these differences were not significant. After controlling for demographics and health characteristics, aripiprazole users reported significantly higher MCS scores (33.91 vs. 31.41, p = .047) and bodily pain (55.06 vs. 46.53, p = .011), general health (49.89 vs. 43.72, p = .034) and emotional role limitation domain scores (49.29 vs. 41.60, p = .017) compared with users of quetiapine (see Figure 4). Differences in health utilities (see Figure 3) and other summary and domain scores were not significant.

Small sample sizes (n = 19, 14, and 11, for olanzapine, risperidone, and ziprasidone, respectively) prevented meaningful comparisons with the other atypical antipsychotics. Aripiprazole users did report significantly greater general health (49.92 vs. 33.93, p = .035) than users of risperidone, however, no other significant differences were observed. In most instances, though not all, the trends were such that higher HRQoL summary and domain scores were observed among users of aripiprazole.

## Discussion

Among patients who self-reported a diagnosis of depression and were using an atypical antipsychotic as part of combination therapy with antidepressants, this study assessed the differences in HRQoL and health utility scores of patients using aripiprazole compared with patients using olanzapine, quetiapine, risperidone and ziprasidone. The results suggest that mental summary, general health domain and mental health domain scores (emotional role limitations and mental health), and overall health utilities are significantly higher among those treated with aripiprazole relative to those treated with other atypical antipsychotics, even after adjusting for demographic and health characteristic differences between these groups. Interestingly, even some physical aspects (i.e., bodily pain) were reported as significantly better for those being treated with aripiprazole. It is important to note that the differences between the groups approached or exceeded clinically-relevant thresholds [17,18].

The results of the current study add to the literature by providing data on head-to-head comparisons of atypical antipsychotics which, other than meta-analyses of clinical trial data and cost studies, are generally lacking. Because of the lack of data, comparisons between the HRQoL effects observed here and in the literature are difficult to make. Overall, the MCS scores reported in the current study were lower than those observed in other studies: 39.0 in Lenert et al. (2000) vs. 34.26 and 32.09 for aripiprazole and other atypical treatments, respectively. This difference is likely due to the fact that respondents in the current study who were being treated with both antipsychotic and antidepressant medications may have had greater depression severity than the average depression population as assessed by Lenert et al. (2000) [4].

As discussed by previous reviews [7,10], limited real-world comparative effectiveness data exist on atypical antipsychotic therapy in MDD. However, clinical trial data generally support the findings observed here. Thase et al. [21] found that a significant reduction in patient-reported functional impairment, as measured by the Sheehan Disability Scale (SDS), was observed among patients with MDD being treated with aripiprazole when compared with placebo [21]. Patients using aripiprazole as augmentation treatment reported lower SDS scores and were significantly more likely to move from ‘severe’ to ‘mild’ and from ‘moderate’ to ‘mild’ levels of impairment [21]. In a meta-analysis of clinical trial data, Nelson and Papakostous [8] found that the efficacy (as measured by response and remission rates) for atypical antipsychotics in MDD was significantly greater than for placebo treatments. Although no statistically significant differences among the atypical antipsychotics were observed, the pooled effects for aripiprazole were higher than other FDA-approved atypical antipsychotics [8].

Studies using real-world data have also suggested a benefit of aripiprazole use as augmentation therapy. A recent study by Jing et al. (2011) suggests that pharmacy costs for patients treated with aripiprazole are higher but total healthcare costs are significantly lower than other atypical antipsychotics primarily due to differences in rates of hospitalization [22]. Further, when comparing FDA-approved atypical antipsychotics with antidepressant monotherapy, aripiprazole had the lowest additional costs per additional responder [23].

### Limitations

Several limitations should be noted from the results of this study. One of the most important limitations is that of selection bias. Although real-world comparative effectiveness data is needed, the disadvantage of such data is that treatments are not randomized. There are likely many factors (e.g., patient characteristics, patient preferences, disease history, etc.) that may have led to the prescription of one atypical antipsychotic over another. These factors may also influence HRQoL. Although an attempt was made to adjust for these differences (prescribing physician, concomitant treatments, years diagnosed, etc.), it is possible other factors not assessed in the analysis could explain the study’s findings. Prospective and longitudinal designs may be beneficial in replicating the effects observed here. It is also important to note that we pooled all other atypical antipsychotics in the main analysis due to small sample sizes. Future research may consider a set of amply-powered individual comparisons to replicate the effects observed here. Given the cross-sectional, observational design of the study, the direction of causality is unknown. Although many alternative explanations for the HRQoL findings have been tested and ruled out (demographics, comorbidities, etc.), it is possible that other unmeasured variables might explain the relationship between depression treatment and the outcomes observed here. The NHWS is a self-reported survey (including medications) so measurement error may have been introduced as it relates to overall group membership (i.e., there was no clinical verification that patients were actually using the medications they reported). It should also be noted that although the NHWS is demographically representative of the overall US adult population, the sample in the current study was restricted by several criteria. It is unknown how the current sample generalizes to the overall MDD population of interest.

## Conclusion

In summary, this study addresses a gap in the knowledge of atypical antipsychotic use as augmentation therapy of patients with MDD. The study results suggest patients taking aripiprazole in combination with an antidepressant in a real-world environment have a statistically and clinically meaningfully higher level of mental quality of life and health utilities than patients taking other atypical antipsychotics in combination with an antidepressant. Although much more research is necessary, particularly utilizing longitudinal designs, this study provides preliminary head-to-head evidence of atypical antipsychotics. These results may help inform real-world effectiveness of these treatments and, as patient outcomes are a valuable part of broader disease management, inform treatment decision making.

## Abbreviations

MDD, Major Depressive Disorder; US, United States; DSM-IV-TR, Diagnostic and Statistical Manual of Mental Disorders (4th Edition Text Revision); HRQoL, Health-related quality of life; SF-`12, Medical Outcomes Study Short Form 12 questionairre; MCS, Mental Component Summary; SF-6D, Medical Outcomes Study Short Form (6 Dimension) health utility; STAR*D, Sequenced Treatment Alternatives to Relieve Depression study; NHWS, National Health and Wellness Survey; BMI, Body Mass Index; FDA, Food and Drug Administration.

## Competing interests

This study was conducted by Kantar Health on behalf of Bristol-Myers Squibb, which funded the study. MD is a full-time employee of Kantar Health. JSW was a full-time employee of Kantar Health at the time of this study and is now a student at Columbia University. IK, JB, and TH are full-time employees at Bristol-Myers Squibb. RF is a full-time employee at Otsuka Pharmaceuitcal Development & Commercialization.

## Authors’ contributions

IK participated in the conception, design, coordination, and analysis of the study. JSW was involved in the coordination and analysis of the study and drafted the manuscript. MD was engaged in the conception and design of the study and participated in the manuscript’s drafting and editing. JB, RF, and TH provided content expertise and editorial insight. All authors read and approved the final manuscript.

## Supplementary Material

Additional file 1 List of medications included in the definition of antidepressant treatment. Click here for file
