# Supplementary material for: Comparison of health-related quality of life among patients using atypical antipsychotics for treatment of depression: results from the National Health and Wellness Survey
Source: Health Qual Life Outcomes. 2012 Jul 17;10:81. doi: 10.1186/1477-7525-10-81 (PMC3411477; doi:10.1186/1477-7525-10-81)
Supplement: Additional file 1 — List of medications included in the definition of antidepressant treatment. [file 1477-7525-10-81-S1.pdf]

Appendix 1: List of medications included in the definition of antidepressant treatment.

Subclass	Medication
Selective serotonin reuptake inhibitors (SSRIs)	citalopram<br>escitalopram<br>fluoxetine<br>fluvoxamine<br>paroxetine<br>sertraline
Serotonin and norepinephrine reuptake inhibitors (SNRIs)	desvenlafaxine<br>duloxetine<br>venlafaxine
Tricyclic antidepressants (TCAs) and related compounds	amitriptyline<br>amitriptyline/chlordiazepoxide<br>amitriptyline/perphenazine<br>amoxapine<br>clomipramine<br>desipramine<br>doxepin<br>imipramine / imipramine pamoate<br>nortriptyline<br>protriptyline<br>trimipramine
Monoamine oxidase inhibitors (MAO-Is)	isocarboxazid<br>phenelzine<br>tranylcypromine
Other antidepressants	nefazodone<br>trazodone<br>mirtazapine<br>maprotiline<br>bupropion SR<br>bupropion XL<br>bupropion
